# Mastoid Abscesses and Their Treatment

**Published:** 1898-09

**Authors:** 


**Mastoid Abscesses and their Treatment.** By A. Broca, M.D., and F. Lubet-Barbon, M.D. Translated and edited by Henry J. Curtis, M.D. Pp. xii., 268. London: H. K. Lewis. 1897.

The preface to this volume states that the work is a translation of a memoir entitled *Les Suppurations de l'Apophyse mastoïde et leur Traitement*, which was published in 1895, after having been awarded the Prix Meynot by the French Academy of Medicine in 1894.

The work is at once scientific and practical. The operations of Schwartze, Stacke, Duplay, and many others are well described and criticised. Clinical notes of no less than 143 cases of mastoid abscesses treated by the authors are introduced into the text of the book, so as to illustrate all the phases of these conditions.

The translation is well done on the whole, though we could have wished that here and there a little more freedom had been exercised in turning the language into English, instead of retaining the original French construction of the sentences. The book gives us in a small compass all the best that is known of the pathology and treatment of mastoid abscesses.

